# An examination of the health and wellbeing of childless women: A cross-sectional exploratory study in Victoria, Australia

**DOI:** 10.1186/1472-6874-11-47

**Published:** 2011-11-10

**Authors:** Melissa L Graham, Erin Hill, Julia M Shelley, Ann R Taket

**Affiliations:** 1 Centre for Health through Action on Social Exclusion (CHASE), School of Health and Social Development, Deakin University, Victoria, Australia

## Abstract

**Background:**

Childlessness among Australian women is increasing. Despite this, little is known about the physical and mental health and wellbeing of childless women, particularly during the reproductive years. The aims of this exploratory study were to: 1) describe the physical and mental health and wellbeing and lifestyle behaviours of childless women who are currently within the latter part of their reproductive years (30 - 45 years of age); and 2) compare the physical and mental health and wellbeing and lifestyle behaviours of these childless women to Australian population norms.

**Methods:**

A convenience sample of 50 women aged between 30 and 45 years were recruited to participate in a computer assisted telephone interview. The SF-36 Health Survey v2 and lifestyle indicators were collected in regards to women's health and wellbeing. Data were analysed using descriptive statistics, t-tests for independent sample means and 95% confidence intervals for the difference between two independent proportions.

**Results:**

Childless women in this study reported statistically significant poorer general health, vitality, social functioning and mental health when compared to the adult female population of Australia. With the exception of vegetable consumption, lifestyle behaviours were similar for the childless sample compared to the adult female population in Australia.

**Conclusions:**

Childless women may be at a greater risk of experiencing poor physical and mental health when compared to the Australian population. A woman's health and wellbeing during her reproductive years may have longer term health consequences and as such the health and wellbeing of childless women requires further investigation to identify and address implications for the provision of health (and other social) services for this growing population group.

## Background

Childlessness is increasing in Australia with 37% of Australian women in their peak reproductive years (30-34) remaining childless in 2006 [1]. The shift in childbearing patterns is creating a need to reflect on this social change and the associated possible negative health and wellbeing consequences which may have implications for policy and service.

Internationally, most health related research on women without children has focused on infertile women or women past reproductive age. A substantial amount of research has examined the physical and mental health of women who are infertile - the involuntarily childless [2-6]. It has been estimated that the natural infertility rate is about 7% and that this has remained stable over time in Australia [7], consequently accounting for only a small proportion of childless women in Australia. As such the physical, mental and social health and wellbeing of the majority of women without children is largely unknown.

Research on childless women who are past reproductive age has demonstrated an association between childlessness and loneliness and depression in older (60 years and over) Chinese women [8] and middle to old age American women [9,10]. Increased psychological distress among childless Canadian women aged over 55 years [11] has also been reported; however, this was in comparison to childless men rather than women with children. Australian longitudinal data suggests that there is no statistically significant difference between older (aged 73-78 years) never married childless women in terms of physical or emotional health, or medical and support service use compared with women currently or previously married women with children [12]. Record linkage data from England and Wales suggests that women less than 60 years of age without children have significantly higher mortality rates compared with women with two children [13]. Women without children have also been found to have an increased risk of breast cancer [14,15], and increased mortality from uterine, ovarian and cervical cancer [15] when compared to women with children. Data from the Japan Collaborative Cohort Study found that childless women aged 40 years or more had a higher risk from all-cause mortality compared to women with children [16]. Data from a prospective British birth cohort study showed that childless women at age 54 were at an increased risk of reporting poor health, which remained after accounting for other socio-demographic variables such as socio-economic status, compared to women who held multiple social roles (mother, wife and strong tie to the labour market) [17]. Survey data from Australia, Finland and the Netherlands of women aged 65 years or more suggests that parenthood has a controlling effect on health risk behaviours such as cigarette smoking, alcohol consumption and physical activity levels, indicating those without children have higher rates of such health risk behaviours when compared to those with children [18].

The limited body of research which has examined the health and wellbeing of childless women during their reproductive years presents conflicting findings. A longitudinal study of men and women in their 30s conducted in the US found that unintended childlessness among women was not associated with psychological distress [19]. However, an Australian cross-sectional study of women aged between 30-34 years suggests that mothers have better mental health than women without children [20]. An Australian longitudinal study of women aged between 22 and 27 and their motherhood intentions found that, compared to women who wanted one or two children, women intending to remain childless were more likely to report poorer social support and depression. Levels of alcohol consumption and overweight and obesity were higher for women who intended to remain childless compared to women who wanted one or two children [21]. Research from Poland which has examined physical activity and positive health behaviours in a group of women aged between 19 to 36 years found that women without children had higher levels of physical activity but were more likely to have addictive behaviours, poorer nutrition and other actions undertaken to maintain health than women with children [22].

Given the limited research which has been conducted on the health status of childless women during their reproductive years, particularly how this compares to women with children, there is an urgent need to examine the health and wellbeing of this growing population group. The evidence suggests that childlessness in later years is associated with significant health and wellbeing consequences which may start during a woman's reproductive years. Identifying and understanding the health and wellbeing of childless women during their reproductive years may enable prevention and/or early intervention strategies to be put in place to reduce the longer term health sequellae. The aims of this exploratory study were to: 1) describe the physical and mental health and wellbeing and lifestyle behaviours of childless women who are currently within the latter part of their reproductive years (30 - 45 years of age); and 2) compare the physical and mental health and wellbeing and lifestyle behaviours of these childless women to Australian population norms.

## Methods

The project was conducted in Victoria, Australia during 2009 and 2010. Human research ethics approval was obtained (141_09) in September 2009.

### Sampling

Convenience sampling was used to recruit a sample of women living in Victoria, of reproductive age (30 - 45 years), who were not currently pregnant and who had never assumed the role of a mother (including biological, adopted, foster or step-children). This age group captures women from the mean age of birth of a first child in Australia (28.2 years) [23] and Victoria (29 years) [24]. As such the sample accounts for the period in a woman's life in which decisions about motherhood intentions are likely to have been formed and enacted.

Women were recruited through a Victorian based university via an email invitation addressed to all female staff. The invitation to participate was open to both academic and non-academic female staff and as such, accounted for a range of socio-economic positions. Women were asked to contact the researchers if they were interested in participating in the study. A plain language statement which detailed the purpose of the study and what participation entailed was sent to all women who responded to the email invitation. The women were then re-contacted a week later to discuss any questions they had about participation in the study and determine their eligibility. If the women consented to participate in the study, a time was made for the computer-assisted telephone interview. Twelve women were excluded as they did not meet the inclusion criteria. The total sample consisted of 50 women.

### Measures

The main variables of interest in this study were divided into three areas: demographic characteristics; physical and mental health; and lifestyle behaviours. Demographic information collected included date of birth, marital status [25], education and income (both individual and household) [26,27]. Physical and mental health and wellbeing were assessed using the SF-36 Health Survey v2 [28]. Australian population norms were obtained for the SF-36 Health Survey v2 [29] and Personal Communication 30 November 2010] to enable comparison between sample scores and the population.

Information on height and weight was collected to calculate Body Mass Index (BMI) [30]. Lifestyle behaviour information collected included physical activity, fruit and vegetable consumption, cigarette smoking and alcohol consumption [26,30]. Population data for BMI and lifestyle behaviours were obtained from 2007-2008 National Health Survey Summary of Results (reissue) [31].

### Data collection

Data were collected using computer-assisted telephone interviews. The interviewer administered the questionnaire and the participant responses were entered into a Microsoft Office Access database created for the purpose of this study. The computer-assisted interviews took approximately 20 minutes to complete.

### Data analysis

Data were transferred from the Microsoft Office Access database to the Statistical Package for the Social Sciences (SPSS) for analysis. Descriptive statistics were used to examine the demographic characteristics of the women, fruit and vegetable consumption, cigarette smoking, alcohol consumption and physical activity. BMI was calculated using the women's height and weight according to the standard formula (weight in kilograms/height in meters^2^). BMI was then grouped as overweight, underweight and normal weight range as defined by the Australian Bureau of Statistics [30] and proportions calculated. Sample proportions were then compared to the Australian data for women only from the 2007-08 National Health Survey [31] and are presented as 95% confidence intervals for the difference in proportions. Data on fruit consumption were categorised as one serve or less and two serves or more per day. Vegetable consumption was categorised as one serve or less, two to four serves or five serves or more per day. Fruit and vegetable data were then compared to the 2007-08 National Health Survey [31] and reported as 95% confidence intervals for the difference in proportions. Comparisons for physical activity and alcohol use could not be undertaken due to differences in reporting of data. Furthermore, given the Australian alcohol guidelines have changed and suggest that no level of drinking is 'safe' or 'no risk' [32] comparison could not be made to the last national health survey which reported alcohol consumption based on the level of "risk".

Scores for the SF-36 Health Survey v2 were computed using the scoring manual as set out by Ware and colleagues [28]; however, Australian norms were provided by Hawthorne and colleagues [29 and Personal Communication 30 November 2010] for females only and were used to calculate t-scores (norm based scores). Means and standard deviations were calculated for each of the eight SF-36 Health Survey v2 domains (General health, Physical functioning, Role physical, Bodily pain, Role emotional, Social functioning, Vitality and Mental health). The sample scores were then compared to the population norms using t-tests.

## Results

### Demographic characteristics

Participant age ranged from 33 to 45 years with the mean age of participants 39.5 (SD = 3.0). Most women were in a relationship with 42.0% of the women currently married. Thirty-two percent of the women were currently not in a relationship. Almost half of the women (46.7%) did not wish to have children and 11.1% identified themselves or their partner as infertile. More than half of the women had a higher university degree (58.0%) and an individual (70.0%) and household (94.0%) income of more than $52,000 per year (Table 1).

**Table 1:** Demographic characteristics of the sample (n = 50)

	Number	Percent
*Marital Status*		
Married	21	42.0
Widowed, divorced or separated	2	4.0
Living with a partner but not married	6	12.0
In a relationship but not living together	5	10.0
Not in a relationship	16	32.0

*Highest level of educational attained*		
Certificate or Diploma	6	12.0
Bachelor level University degree	15	30.0
Higher university degree (e.g. Grad Dip, Masters, PhD)	29	58.0

*Individual Income*		
Less than $52,000 annually	15	30.0
$52,000 or more annually	35	70.0

*Household income*		
Less than $52,000 annually	3	6.0
$52,000 or more annually	47	94.0

### Physical and mental health

Compared to one year ago, more than half (54.0%) of the women reported that in general their health was about the same, 22.0% reported their health was now somewhat better, 16.0% reported that their health was much better now and 8.0% reported that their health was worse than one year ago. Women in this study reported statistically significant poorer general health (-10.3; 95%CI -13.2 - -7.3), vitality (-4.9; 95%CI -7.7 - -2.1), social functioning (-16.3; 95%CI -19.3 - -13.3) and mental health (-7.2; 95%CI -10.2 - -4.3) when compared to the female population (Table 2).

**Table 2:** SF-36 Health Survey v2 (n = 50)

Dimension	Percentage Scores		T-Scores		
	Sample Mean (SD)	Population norm (SD)^2 ^	Norm based sample mean (SD)	Population norm (SD)^3 ^	Difference in mean and 95%CI
Physical functioning	95.9 (6.75)	84.64 (21.86)	55.15 (3.09)	48.18 (11.31)	6.97 (3.83 - 10.11)
Role physical^1^	91.33 (11.89)	84.41 (25.13)	52.75 (4.73)	49.13 (10.52)	3.62 (0.66 - 6.58)
Bodily pain	73.64 (20.45)	76.45 (21.24)	48.68 (9.63)	48.95 (10.27)	-0.27 (-3.16 - 2.62)
General health	48.48 (10.94)	71.90 (21.88)	39.30 (5.00)	49.58 (10.57)	-10.28 (-13.22 - -7.34)
Vitality	47.13 (9.21)	61.12 (20.80)	43.27 (4.43)	48.16 (10.21)	-4.89 (-7.73 - -2.05)
Social functioning	47.50 (8.38)	86.19 (22.33)	32.67 (3.75)	48.97 (10.74)	-16.30 (-19.28 - -13.32)
Role emotional	91.00 (16.13)	91.59 (17.50)	49.66 (9.22)	49.07 (11.08)	0.59 (-2.52 - 3.70)
Mental health	66.30 (8.44)	80.63 (16.99)	41.57 (4.97)	48.81 (10.46)	-7.24 (-10.15 - -4.33)
Physical component summary score^1^			51.01 (4.28)	48.76 (11.07)	2.25 (-0.86 - 5.36)
Mental component summary score^1^			39.19 (5.74)	49.08 (10.75)	-9.89 (-12.92 - -6.86)

^1 ^n = 49

^2 ^Both males and females aged 18+

^3 ^Females aged 18+ only

### Lifestyle behaviours of childless women

The women's BMI ranged from 19.2 to 40.4 with a mean BMI of 26.5 (SD = 5.3). Almost half (45.8%) of the women's BMI was within normal range, 33.3% were overweight and 20.8% were obese. Table 3 shows the women's BMI in comparison to the population norms for Australian women and suggests that there was no statistically significant difference between the study sample and population in relation to BMI. Almost half (48.0%) of the women only consumed one serve or less of fruit per day, but there was no statistically significant difference between the study sample and the population. There was a statistically significant difference in the proportion of women who ate five or more serves of vegetables per day between the study sample (22.0%) and the population (10.1% 95%CI for difference 2.6 - 25.1). Most women had never smoked cigarettes (56.0%) with about one-third of the women ex-smokers (34.0%) and 10.0% current smokers. Of the women who currently smoked cigarettes, 80.0% smoked daily and 20.0% smoked weekly (at least once a week but not daily). There was no statistically significant difference between women in the study sample and the population (Table 3).

**Table 3:** BMI, fruit and vegetable consumption and smoking status (n = 50)

	Percent (n)	2007-08 National Health Survey^1 ^	Difference in proportion and 95% CI
*BMI^2^*	*n = 48*	*n = 5,586*	
Normal weight	45.8 (22)	42.6 (2,380.3)	3.22 (-10.1 - 17.16)
Overweight	33.3 (16)	31.0 (1,734)	2.29 (-9.43 - 16.47)
Obese	20.8 (10)	23.6 (1,320.4)	-2.8 (-10.67 - 11.98)

*Fruit^3 ^*	*n = 50*	*n = 8,412.8*	
One serve or less	48.0 (24)	39.0 (3,280.4)	9.01 (-4.24 - 22.54)
Two or more serves	52.0 (26)	56.4 (4,743.3)	4.38 (-8.86 - 17.91)

*Vegetables^4^*	*n = 50*	*n = 8,412.8*	
One serve or less	18.0 (9)	23.1 (1,942.7)	-5.09 (-7.73 - 13.37)
Two to four serves	60.0 (30)	66.2 (5,565.9)	-6.16 (-6.27 - 20.01)
Five or more serves	22.0 (11)	10.1 (853.6)	11.85 (2.58 - 25.11)

*Smoking status *	*n = 50*	*n = 8412.8*	
Current smoker	10.0 (5)	18.0 (1,514.7)	-8 (-3.38 - 13.72)
Ex-smoker	34.0 (17)	24.3 (2,042.1)	9.73 (-1.87 - 23.6)
Never smoked	56.0 (28)	57.7 (4,856)	-1.72 (-11.16 - 15.46)

^1 ^Weighted sample.

^2 ^Underweight has been excluded from analysis as no women in the sample were underweight.

^3 ^Don't eat fruit has been excluded from analysis as this was not reported by any women in the sample.

^4 ^Don't eat vegetables has been excluded from analysis as this was not reported by any women in the sample.

The mean number of times in the last two weeks the women walked (for sport, recreation or fitness) was 5.7 (SD 4.0). The mean number of times in the last two weeks the women undertook moderate physical activity was 4.9 (SD 3.8) and vigorous physical activity was 3.3 (SD 3.6).

Only a small percentage of women did not drink alcohol (6.0%) with about one-third (34.0%) of women drinking alcohol on one or two days per week. Of the women who did drink alcohol, 23.4% of women had not drank in the last week and 29.8% had drank once, 40.4% consumed five standard drinks or more on one occasion less than once a month and 65.2% usually drank one or two standard drinks per day on a day when they drank alcohol.

## Discussion

The current study has examined the physical and mental health and wellbeing in a sample of childless women during the latter part of their reproductive years and how their health and wellbeing compares to available Australian population norms. The findings suggest that these childless women experience significantly poorer general health, vitality, social functioning and mental health when compared to the Australian female population. These findings are important because they suggest that childlessness may have significant health implications for women during their reproductive years. Holton and colleagues (2010) also found that women without children reported poorer mental health than mothers. Similarly, Lee and Gramotnev (2006), in their study of motherhood intentions, found that young women who intended to remain childless reported significantly poorer mental health compared to young women who intended to mother [21]. However, it must be noted that Lee and colleagues assessed health outcomes based on current childless women's intentions for motherhood rather than childlessness itself. Evidence suggests that childless women are marginalised, stigmatised and socially excluded [33-38] and that the health status of such population groups is poorer than the general population [39], thus potentially explaining why childless women experience poorer health.

The lifestyle behaviours of these childless women, in general, were similar to those for Australian females with the exception of vegetable consumption. Childless women in the present study were more likely to consume five or more serves of vegetables per day compared to the Australian female population. These findings differ to those reported by others who suggest that lifestyle behaviours such as cigarette smoking and fruit and vegetable consumption are poorer for childless women when compared to women with children, suggesting that parenting has a controlling effect on health risk behaviours [18,22]. It is possible that the difference in the study findings is due to how lifestyle behaviours were measured, the sample age, socio-economic status or employment status, or the study sample size.

Past research suggests that physical activity levels are higher in women who do not have children compared to women with children [22]; however, this study was not able to confirm this finding as it was not possible to compare sample data to population data for Australian females. It has also been suggested that childlessness is associated with high levels of alcohol consumption [21,22]; however, this study was not able to investigate childless women's level of alcohol consumption in comparison to population data due to differences in data reporting and changes to Australian alcohol guidelines. Australian alcohol guidelines now warn that there is no 'safe' level of alcohol consumption [32] and therefore previous national surveys [30,31] which describe alcohol consumption in terms of 'risk' could not be used for comparison. However, based on the previous Australian alcohol guidelines and findings from earlier research [21] it is likely that these childless women are at a much greater risk of unsafe levels of alcohol consumption. In any case, based on this study's findings, the association between childlessness and lifestyle behaviours warrants further examination.

Although this pilot project has provided valuable insight into these childless women's physical and mental health and wellbeing, it is not without its limitations. This pilot study was based on a small convenience sample of childless women in Victoria, Australia, thus these findings must be considered with caution and cannot be generalised to the wider population of childless women. Accordingly, the physical and mental health and wellbeing of the women could not be compared by the types of childlessness. However, it is possible that there are physical and mental health and wellbeing differences between the types of childlessness [40], which are masked in the current analysis.

The current study found that more than half of the women were not currently married, with 32% of the women currently not in a relationship. Similarly, data from the Australian Bureau of Statistics suggest that in 2001 32% of the Australian population aged 15 years or more had never been married. Over the past 20 years in Australia, there has been a decline in marriage rates along with an increase in the number of women never marrying [41]. However, there has been an increase in the number of babies born outside registered marriages and an increase in the number of people living in de facto relationships. Previous research suggests that childlessness is influenced by marital status [41-43] with the unmarried more likely to remain childless. Marital status may influence a woman's health and wellbeing however due to the small sample size it was not possible to test for associations between marital status and physical and mental health and wellbeing.

Furthermore, the sample was based on a sample of university employees (both academic and general staff) and thus is not representative of all childless women. Further to this, the sample obtained was biased towards higher socio-economic status based on education and income. As such the health and wellbeing of childless women reported here may not be reflective of all childless women across the diversity of socio-economic positions.

Given the cross-sectional nature of this study it is not possible to examine temporality - is childlessness the result of poor health and wellbeing or does childlessness cause poor health and wellbeing? Longitudinal studies are required to further investigate these findings and any potential cause and effect relationships. Despite these limitations, this pilot study has a number of strengths. Namely, it has examined childless women's health and wellbeing in the context of their reproductive lives which enables the early identification of potential health risks which may remain into older age.

## Conclusions

Childless women may be at a greater risk of experiencing poor physical and mental health when compared to the Australian population. The observed differences in this study may be the result of this study's lack of power or the nature of the convenience sample. Regardless, these findings require further investigation to determine the health and wellbeing of childless women in their reproductive years. A woman's health during her reproductive years may have longer term health consequences. There are implications for the provision of health (and other social) services for this growing population group which need to be identified and addressed. If women without children report poorer health in later life then we need to identify 'at risk' women during their reproductive years to allow for early intervention. This will enable the development and implementation of appropriate and relevant public health strategies which can be targeted to these women to address current poor health as well as prevent later poor health.

## Competing interests

The authors declare that they have no competing interests.

## Authors' contributions

MG conceived the study, developed the instrument and data collection methodology, undertook the data analysis and prepared the manuscript. EH collected the data and contributed to the data analysis and manuscript preparation. JS and AT provided input into the instrument development, data analysis and manuscript preparation. All authors read and approved the final manuscript.

## Pre-publication history

The pre-publication history for this paper can be accessed here:

http://www.biomedcentral.com/1472-6874/11/47/prepub
